# Seasonal impact on the thermal tolerance window of a keystone coastal grazer, the common periwinkle *Littorina littorea*

**DOI:** 10.1007/s00360-026-01670-3

**Published:** 2026-05-29

**Authors:** Tomás A. García, Daria Bedulina, Inna Sokolova, Gisela Lannig

**Affiliations:** 1 Department of Integrative Ecophysiology, Alfred-Wegner-Institute Helmholtz Centre for Polar and Marine Research, Bremerhaven, Germany; 2 Department of Marine Biology, Institute for Biological Sciences, University of Rostock, Rostock, Germany; 3 Department of Maritime Systems, Interdisciplinary Faculty, University of Rostock, Rostock, Germany

**Keywords:** Gastropod, Warming, Seasonal acclimatization, Behavioral response, Metabolomics, Oxidative stress

## Abstract

Temperature influences the performance of marine ectotherms, and understanding species’ thermal responses is key to predicting climate change impacts. This study investigated seasonal acclimatization effects on the thermal tolerance of the common periwinkle *Littorina littorea*. Snails were collected in winter and summer on the North Sea Island of Helgoland and exposed to gradually increasing temperatures (1.5 °C/h from the sampling temperature, 10–16 °C to 34 °C). Behavioral responses (antenna extension and movement onset) were assessed at target temperatures (10/16, 22, 25, 28, 31, and 34 °C), alongside cellular responses measured via activity of catalase (CAT), Hsp70 content and ^1^H NMR-based metabolite profiling. Both winter and summer groups showed delayed behavioral responses above 22 °C, indicating a similar behavioral breakpoint temperature across seasons. Metabolite data revealed temperature-dependent changes only in the winter group, with elevated succinate, glutathione, glutamine and propionate levels at 28 °C, indicating anaerobic metabolism and onset of critical thermal limits. While the upper suboptimal temperature appeared similar between groups (22 °C), the upper critical temperature (Tc) was detected only in the winter group (28 °C). Summer snails had significantly higher baseline Hsp70 levels and slightly higher CAT activities, indicating enhanced cellular protection in conjunction with a seasonal shift in Tc. Overall, the results show that seasonal acclimatization alters acute thermal responses in *L. littorea* and behavioral metrics are confirmed as valuable sublethal indicators for assessing heat tolerance in marine invertebrates.

**Graphical abstract:**

**Figure:**
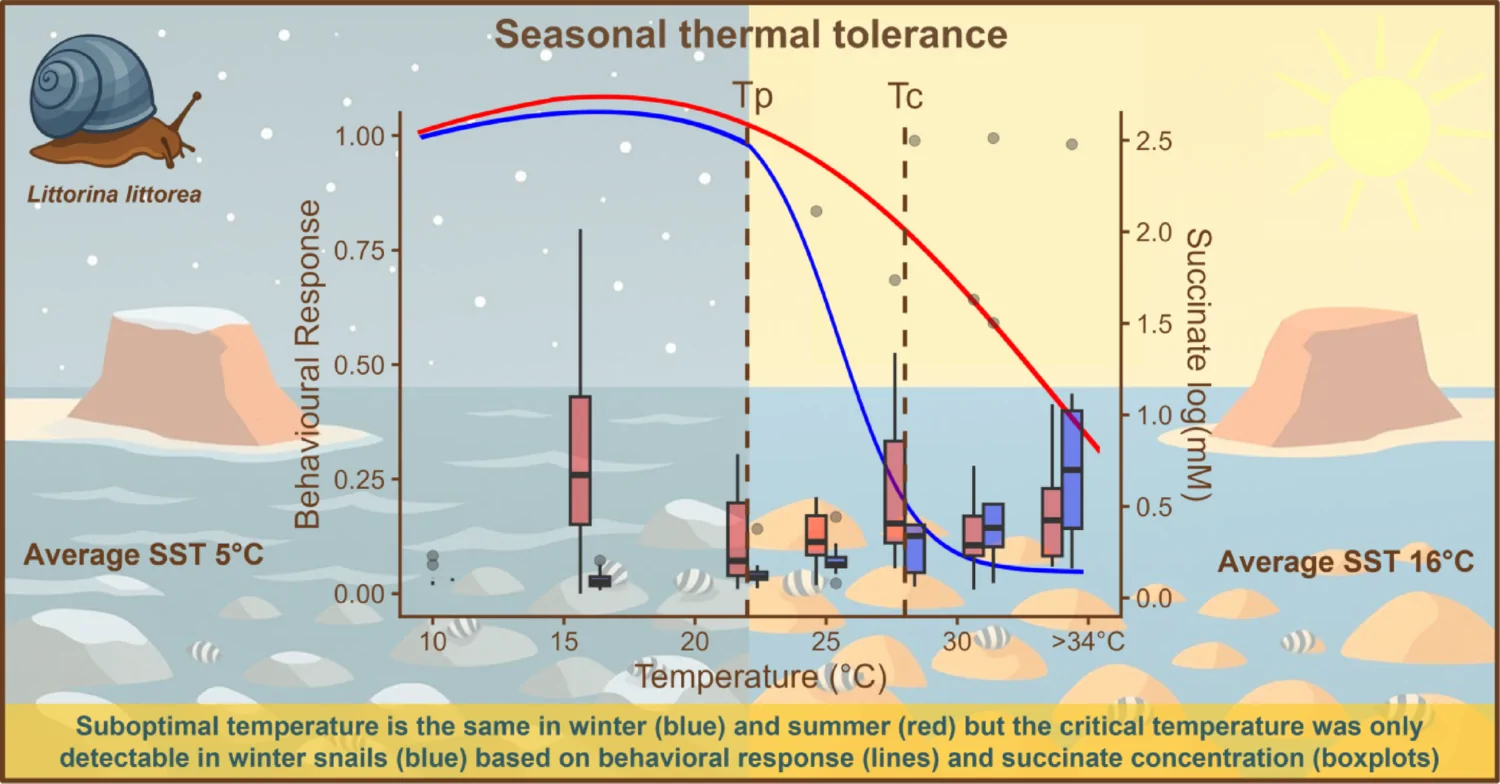

**Supplementary Information:**

The online version contains supplementary material available at 10.1007/s00360-026-01670-3.

## Introduction

Temperature influences biological systems at all levels of biological organization, from molecules to entire ecosystems, and serves as a major factor influencing the structure and function of marine ecosystems. Understanding the response of marine ectotherms to temperature change is thus a key factor for predicting the effects of climate change on marine ecosystems (Rose et al. 2024). Over the past century, oceanic temperatures have increased (IPCC 2023), with projections indicating continued warming and an increased frequency of extreme weather events, such as marine heatwaves (Kaiser et al. 2023). These changes are particularly concerning for rocky intertidal zones, which are characterized by extreme physical heterogeneity and sharp fluctuations in abiotic factors such as wave action, irradiation, salinity, and temperature (Johannesson 2003). Studies on intertidal organisms have shown that they live close to the thermal limits of their physiological tolerance, and even relatively small shifts in temperature may threaten their performance and survival (Tomanek and Helmuth 2002). Among the key species inhabiting rocky shores is *Littorina littorea* (Linnaeus, 1758), a common periwinkle found in marine intertidal zones from the Mediterranean to the North Sea (Lillebjerka et al. 2023). It mainly occurs in the low and mid-tidal zones, including tide pools (Yamada and Mansour 1987; Titmuss et al. 2025) where it can reach densities of up to hundreds of individuals per square meter, such as on the islands of Helgoland and Sylt in the south-eastern part of the North Sea (Eschweiler et al. 2008). *L. littorea* exerts a moderating grazing pressure on intertidal macroalgae, influencing rocky shore succession and maintenance, and controlling both seasonal and perennial algal growth and settlement (Janke 1990) as well as community structure (Yamada and Mansour 1987; Brenchley and Carlton 1983). Moreover, due to their grazing activity, *L. littorea* breaks down pieces of seaweed, making it available as food for smaller organisms, increasing nutrient release, and surface area for microbial colonization, contributing to carbon turnover (Lillebjerka et al. 2023). Aside from the ecological importance of *L. littorea*, this species is considered a delicacy in certain parts of Europe, and in the early 2000s, 60,000 tonnes per year were harvested (Lillebjerka et al. 2023). Despite the ecological and economic relevance of *L. littorea*, the thermal tolerance of this species remains poorly studied.

Early studies investigated extreme cold or heat tolerance, including freezing resistance (Murphy and Johnson 1980) and mortality or heat-coma temperatures under acute exposures (Fraenkel 1960; Clarke et al. 2000). In 1960, Fraenkel studied the heat tolerance by measuring the mortality of *L. littorea* after a recovery period (24 and 48 h) following exposure (up to 330 min (5.5 h) dependent on exposure temperature) to temperatures between 39 °C and 41 °C (Fraenkel 1960). After 330 min of exposure at 39 °C, about 50% of the animals died, while the remaining individuals fully recovered. In comparison, approximately 50% mortality occurred after 90–120 min at 40 °C and after only 60 min at 41 °C. By increasing the temperature by 1 °C/5 min, Clarke et al. (2000) determined the heat-coma temperature (temperature at which the pedal muscle can no longer remain attached to a surface) of *L. littorea* at about 31 °C. The broad thermal tolerance observed in *L. littorea* is consistent with its adaptation to the highly variable intertidal environment (Johannesson 2003; Titmuss et al. 2025). However, these approaches provide only coarse estimates of tolerance limits and give limited insight into sublethal thresholds, which affect fitness-related functions such as behaviour, growth, and metabolism and can therefore determine the long-term viability of populations (Pörtner and Farrell 2008; Morell et al. 2024). While some studies have examined such thresholds in early life stages of *L. littorea*, for example Lillebjerka et al. (2023) who reported a suitable temperature range for embryonic development of 7–20 °C, the thermal sensitivity of adults and the extent to which temperature thresholds shift due to physiological plasticity remain poorly understood.

Based on the extensive research of thermal tolerance range in ectotherms, the concept of Oxygen- and Capacity-Limited Thermal Tolerance (OCLTT) has been developed to delineate temperature thresholds by linking specific physiological responses with temperature changes (Pörtner 2001, 2010), and has received considerable support for its applicability in aquatic organisms (Angeles-Gonzalez et al. 2023; Baag et al. 2020; Diaz et al. 2021; Diederich and Pechenik 2013; Gibson and Harris 2023; Meza-Buendía et al. 2021; Sokolova 2013) while also being the subject of ongoing debate (Clark et al. 2013; Jutfelt et al. 2018). The OCLTT proposes that an organism’s thermal tolerance is determined by its ability to maintain aerobic energy metabolism and integrates mechanisms such as anaerobiosis and heat shock response that allow the organism to temporarily tolerate temperature extremes (Pörtner 2001, 2021). The concept defines species-, population-, and season-specific active and passive thermal tolerance ranges delineated by temperature thresholds, the pejus (Tp) and critical (Tc) temperatures. The optimal thermal range for steady-state routine performance such as growth is limited by so-called pejus (“pejus”—“getting worse”, lat.) temperatures. With warming, the upper Tp defines the onset of capacity limitation and hypoxaemia as (venous) oxygen partial pressure (P_O2_) progressively declines outside the optimal thermal range, signaling a deterioration in oxygen supply. The upper Tc finally denotes the transition to anaerobic metabolism to fuel energy demand which is no longer met by aerobic energy production due to limited oxygen supply via impaired ventilatory and/or circulatory capacities (Pörtner and Lannig 2009; Pörtner et al. 2017; Pörtner 2021). In the context of climate change, sublethal temperature thresholds, such as those provided by the OCLTT framework, represent a more ecologically meaningful approach to assessing thermal stress than methods that focus solely on lethal limits, as they provide early warning signals for ecologically-relevant physiological disturbances (Rose et al. 2024). For example, Ángeles-González et al. (2020) and Ángeles-González et al. (2021) used sublethal temperature thresholds to determine the distribution of octopus species under climate change. Identifying sublethal thermal thresholds, such as Tp, is therefore essential for predicting the vulnerability and resilience of populations under future climate scenarios. This is particularly relevant for key intertidal species such as *L. littorea*, as failure to reproduce or maintain energy balance may occur during increasingly frequent heat events, even in the absence of mortality. Seasonal variation further complicates the assessment of thermal limits as metabolic adjustments, including changes in membrane composition (Gabbianelli et al. 1996; Facchini et al. 2018), enzyme activity (Kong et al. 2008; Gunay et al. 2020), and/or mitochondrial function (Keller et al. 2004; Sommer and Pörtner 2004), reshape thermal windows in marine ectotherms (Sommer et al. 1997; Wittmann et al. 2008; Jost et al. 2015; Zhang et al. 2021; Peck et al. 2014; Hofmann and Todgham 2010; Pörtner et al. 2017).

The present study aims to address knowledge gaps in the thermal tolerance window of *Littorina littorea* in the context of the OCLTT framework, taking into account seasonal influences. Specifically, the aim was to determine the upper sublethal thermal limits—pejus (Tp) and critical (Tc) temperatures—of a North Sea population collected in winter and summer. We expect distinct physiological changes at each threshold: at Tp, a decline in overall physiological performance reflecting the onset of aerobic capacity limitation; and at Tc, a transition to anaerobic metabolism and the activation of general cellular stress responses. To test that, we measured behavioural responses to assess changes in the animals’ overall performance, metabolite profiles to assess changes related to energy metabolism, the neuroendocrine system, osmolarity and amino acid metabolic processes, and two common markers for cellular stress response—levels of heat shock proteins (Hsp70), and the activity of the antioxidant enzyme catalase. Based on the concept of seasonal acclimatization and increased thermal tolerance in summer, we hypothesised that the winter group would exhibit higher thermal sensitivity and a lower sublethal thermal thresholds compared to the summer group.

## Methodology

### Animal collection and maintenance

*Littorina littorea* was collected from the intertidal zone at Helgoland Südstrand in the North Sea (54°11′ N, 7°53′ E) during two seasons: November 2023 (autumn/winter) with a sea surface temperature (SST) of 9 °C and June 2024 (summer; SST = 16 °C). Helgoland has a temperate marine climate with mean sea surface water temperatures (1982–2023) of 5.6 °C in winter (December-February), 16.1 °C in summer (June-September), and 10.6 °C annually (SST data from the Daily Gridded Optimum Interpolation Sea Surface Temperature (OISST) from the ERDDAP server of the US National Oceanic and Atmospheric Administration (https://coastwatch.pfeg.noaa.gov/erddap/griddap/ncdcOisst21Agg_LonPM180.html, last accessed 2024-03-01). Adult snails (average shell size = 13.7 mm) were kept cool and transported to the Alfred Wegener Institute (AWI) in Bremerhaven, Germany. The snails were kept in the aquarium system with filtered, recirculating and continuously aerated seawater with salinity of 33 and fed ad libitum with brown macroalgae (*Fucus vesiculosus*) collected from Helgoland. Acclimation temperatures depended on the collection season: winter snails at 10 °C and summer snails at 16 °C.

### Experimental setup

To determine the upper pejus and critical temperatures of *L. littorea*, 140 snails of similar size (13 ± 1 mm) and mass (3.88 ± 0.60 g) were divided into two 20-litre aquaria. Each aquarium was filled with the same seawater used during acclimation and placed in a 150-liter Coleman cooler filled with seawater. Temperature control was achieved using a LAUDA Eco Gold Immersion Thermostat (LAUDA, Germany) with a 1:1 mixture of glycogen and distilled water as the cooling medium. After transfer to the experimental system, snails were allowed to rest for 24 h at the season-specific acclimation/control temperature (10 °C for winter and 16 °C for summer) (Clarke et al. 2000) to minimize handling effects. The temperature was then increased at a rate of 1.5 °C/h, following the method of Sokolova and Pörtner (2003) for *L. saxatilis* from the Helgoland region. The temperature ramp was terminated at 34 °C, as heat coma is reported for *L. littorea* at 35 °C (Clarke et al. 2000). Throughout the experiment, the water was continuously mixed by submersible pumps (ProFlow t500; JBL GmbH & Co. KG, Germany) and aerated to maintain oxygen levels, ensure proper circulation, and prevent vertical temperature gradients. Water temperature was monitored every 5 min using a fiber-optic thermometer (FOTEMP1-4; Weidmann Optocon, Germany).

**Fig. 1:**
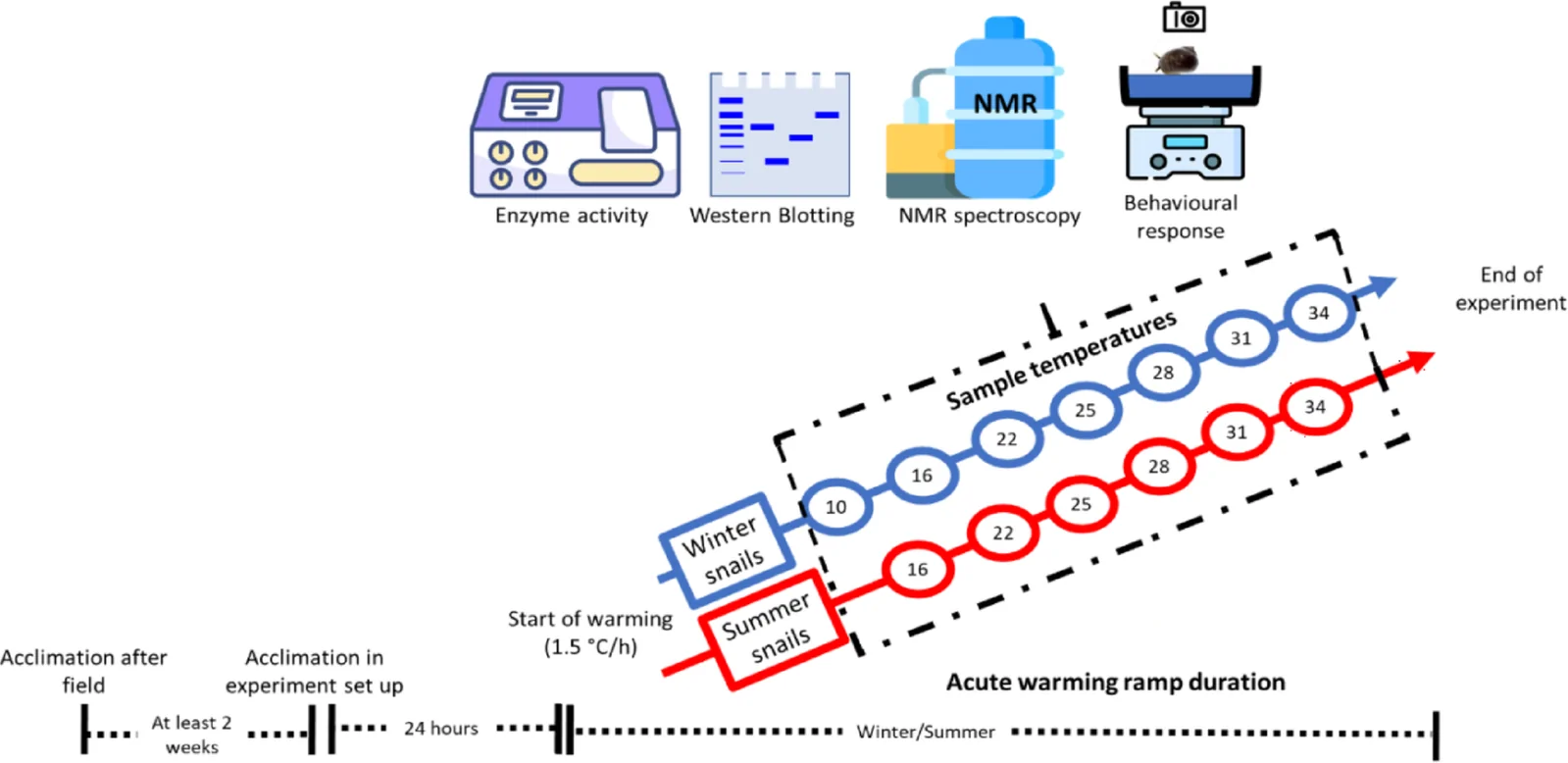
This is a schematic representation of the experimental design. The bottom dashed lines represent the time duration in each step before and during the acute winter and summer warming ramps. Both ramps were performed at the same warming rate (1.5 °C/day), and the sampling temperatures are shown as blue (winter group) and red (summer group) circles in the middle section. The sampled snails were analyzed using each of the four methods described in the top section of the figure

At specific target temperatures, 20 snails in total were randomly selected to measure behaviour (*N* = 10) and cellular response (*N* = 10, see below). Sampling temperatures were as follows: winter snails − 10, 16, 22, 25, 28, 31, and 34 °C; summer snails − 16, 22, 25, 28, 31, and 34 °C (Fig. 1) and are based on literature references to *Littorina saxatilis* from Helgoland (Tc 28 °C, Sokolova and Pörtner 2003), with a low sampling interval (every 3 °C) around 28 °C to fully observe the changes in the organisms and more precisely determine the pejus (Tp) and critical temperatures (Tc).

### Behavioural response

The behavioral response was monitored using videorecording according to the method described by Alonso (2023). Snails (*N* = 10) were transferred to a white plastic plate immersed in a water bath at each of the target temperatures (winter snails − 10, 16, 22, 25, 28, 31, and 34 °C; summer snails − 16, 22, 25, 28, 31, and 34 °C). To avoid thermal fluctuations during the transfer, the snails were placed in beakers filled with water at the same temperature from which they were transferred directly onto the plastic plates. The plate contained equally sized squares (15 × 5 cm) filled with seawater to a depth of 2 mm. Snail behavior was recorded with camera A39 (Hoestr, Germany) for 15 min, and the following endpoints were determined: (1) Time to antenna extension after transfer, and (2) Time to start movement. After behavioral recording, the snails were removed from the experiment and transferred to acclimation aquariums. No snail was used to measure behavioral responses at more than one temperature.

### Cellular response

Snails that were not used for the behavioral endpoints were sampled for the measurement of the cellular response. The snails were blotted dry, wrapped in aluminum foil, and immediately frozen and stored in liquid nitrogen for later analysis. For tissue extraction, shell material was removed under liquid nitrogen and frozen snail’s soft body was ground using a Spex SamplePrep 6775 Freezer/Mill (Spex SamplePrep, USA) with liquid nitrogen as a cooling medium for two grinding cycles of 1 min each and 15 cycles per second. Powdered soft body samples were stored in cryogenic tubes at − 80 °C until further analysis.

### Metabolite analysis

Metabolites were extracted from the whole soft body of *L. littorea* using a modified methanol-dichloromethane protocol (Tripp-Valdez et al. 2017). Briefly, 100 mg of powdered tissue was homogenized in 450 µL ice-cold methanol and 140 µL Milli-Q water using a Precellys 24 homogenizer (Bertin Technologies, France) at 6000 rpm (2 cycles, 20 s each, 0–4 °C). After homogenization, 450 µL of dichloromethane was added and the samples were incubated on ice for 15 min. The aqueous and organic phases were separated by centrifugation (3000*g*, 4 °C, 15 min). The aqueous phase was collected, dried in a SpeedVac concentrator overnight (RVC 2–33 IR, Christ GmbH, Germany), and resuspended in a 1:1 ratio of tissue and deuterated water (D₂O) containing trimethylsilyl propionate (TSP, 0.05 wt%, Sigma-Aldrich 450510) as an internal standard, resulting in a final concentration of 3.2 mM. Measurements were performed as described in Bedulina et al. (2024) using an ultra-shielded vertical 9.4 T NMR spectrometer (Advance III HD 400 WB, Bruker-BioSpin GmbH, Germany), with a 1.7 mm triple tuned ^1^-^13^ C-^15^N NMR probe and a Carr-Purcell-Meiboom-Gill sequence (Bruker protocol cpmgpr1d, TOPSPIN 3.5, Bruker GmbH, Germany) with water suppression. The applied parameters were as follows: acquisition time (AQ) = 4.01 s, sweep width (SWH) = 8802 Hz (22 ppm), delay (D1) = 4 s, dummy scan (DS) = 4, and number of scans (NS) = 128. Spectra processing and analyses were done with Chenomx NMR Suite 8.4 software (Chenomx Inc., Edmonton, Canada) whereby the spectra were corrected for phase, shim and baseline and calibrated to the internal TSP signal (at 0.0 ppm, 3.2 mM). Metabolites were identified using the internal database of Chenomx and an in-house library of ^1^H NMR spectra from marine invertebrates. Due to the difficulties with differentiation of the signals from AMP, ADP and ATP, signals were summed and are reported as adenylates.

Heat Shock Proteins.

Significant accumulation of Hsp70 protein in ectotherms is typically observed when individuals are exposed to temperatures approaching their upper thermal limits (Tomanek and Somero 1999). Therefore, for comparison between winter and summer groups, we focused on the maximum exposure temperature (34 °C) and the respective control temperatures (10 and 16 °C). Hsp70 levels were determined by immunoblotting following protein separation by sodium dodecyl sulfate-polyacrylamide gel electrophoresis (SDS-PAGE) following the protocols of Laemmli (1970) and Towbin et al. (1979) with modifications, described in Bedulina et al. (2017). Powdered snail tissue was homogenized 1:3 in 0.1 M Tris-HCl buffer (pH 7.6) with protease inhibitor cocktail (Sigma P8340; final concentration 1%) on ice and centrifuged at 7000 g for 15 min at 4 °C. The supernatant was mixed with Laemmli sample buffer (0.0625 M Tris-HCl, 1 mM EDTA, 1% SDS, 20% glycerol, 5% β-mercaptoethanol, and 0.001% bromophenol blue, pH 6.8). Total protein concentration was determined by the Bradford assay (Bradford 1976). Equal amounts of protein were separated by SDS-PAGE in 10% polyacrylamide gels using a Mini-PROTEAN 3 electrophoresis system (BIO-RAD, USA). Western blotting was performed via wet transfer using the same Mini-PROTEAN 3 electrophoresis system (BIO-RAD, USA). Equal loading of protein was verified by staining membranes with 0.5% Ponceau Red in 1% acetic acid. After blotting, the membranes were blocked in 5% non-fat dry milk solution (Biomol). Hsp70 determination was performed using a monoclonal anti-HSP70 AB primary antibody (Sigma #H5147) that is designed to detect conservative domains of various species. This antibody was previously used in amphipods (Bedulina et al. 2013) and gastropods (Axenov-Gribanov et al. 2014). The antibody was used at 1:5000 dilution in a blocking solution for 22 h, followed by incubation with an alkaline phosphatase-conjugated secondary antibody (anti-mouse IgG: AP Conj., Cytiva NA931V) at 1:1000 dilution for 2 h. The membranes were then incubated with the chemiluminescent signal ECL prime western blotting detection reagent (Cytiva RPN2236), and the protein bands were visualized with the LAS-1000 (Fujifilm, Japan) imaging system (see supplementary Fig. 7). The signal intensity was quantified with ImageJ software with the “Fiji” package (Schneider et al. 2012), using the add-on (Gurkov et al. 2014). An integrative control sample (a pooled sample from all individuals) was included on each gel and used as an internal reference. Hsp70 levels were expressed in arbitrary units relative to this control, providing a baseline for comparison with samples at control temperatures and 34 °C.

### Catalase activity

Enzyme extraction from the whole soft body of *L. littorina* was performed as described by Bedulina et al. (2010). Briefly, ground tissue (~ 20 mg) was homogenised by a micropestle in three volumes of ice-cold 0.1 M sodium phosphate buffer (pH 6.5) including protease inhibitor cocktail (Sigma P8340; final concentration 1%) and centrifuged at 10,000*g* and 4 °C for 3 min. Using a Synergy H1 microplate reader (BioTek, USA), catalase activity (CAT) of the supernatant was measured spectrophotometrically at 240 nm, using 2.25 mM hydrogen peroxide as a substrate and 25 °C according to Aebi (1984). CAT activity was expressed in U/mg (µmol per min per mg of protein) based on protein determination after Bradford (1976).

### Statistical analysis

Metabolite data were analyzed using the MetaboAnalyst 6.0 web application (Xia and Wishart 2016; Bock et al. 2024). Data were log₁₀-transformed and pareto scaled. Significance Analysis of Microarrays (SAM) was performed with the delta value adjusted to maintain a false discovery rate (FDR) below 0.05. Partial Least Squares Discriminant Analysis (PLS-DA) was applied, with a variable importance in projection (VIP) score above 1 considered indicative of relevant discrimination. Classification and cross-validation by permutation tests based on separation distances were used to assess potential overfitting and evaluate the validity of the PLS-DA model. Additionally, pathway analyses were conducted as described in Götze et al. (2025). Metabolites from the respective control groups (winter: 10 °C; summer: 16 °C) were compared against each of the higher temperature conditions. The analysis settings were as follows: global test as the enrichment method, relative-betweenness centrality for topology analysis, and the zebrafish *Danio rerio* reference metabolome library. Pathways containing more than 30 compounds but only one assigned metabolite with a negative common logarithm of the p-value below 2.5, and those with an impact score below 0.1 were excluded.

Other statistical analyses were performed in R version 4.4.2 (R Core Team 2024). Selected metabolites based on SAM and PLS-DA results, behavioural responses and CAT activity data were tested using the Kruskal–Wallis test (with Dunn’s post hoc tests) to determine differences between temperatures within each season. Additionally, a Wilcoxon rank-sum test was used to assess the interaction effects between temperature and season and to account for multiple comparisons we applied the Benjamini–Hochberg false discovery rate correction. For Hsp70, differences in means between and within each group were tested with a T-test. Breakpoint temperatures for behavior (BBT) and metabolite concentrations (MTB) were determined using two-segment linear regression described by (Muggeo 2003, 2008) and a David test to determine if there are significant changes in the slopes of the linear regressions. Statistical significance for mentioned tests was set at *p* ≤ 0.05.

## Results

### Behavioral response

**Fig. 2:**
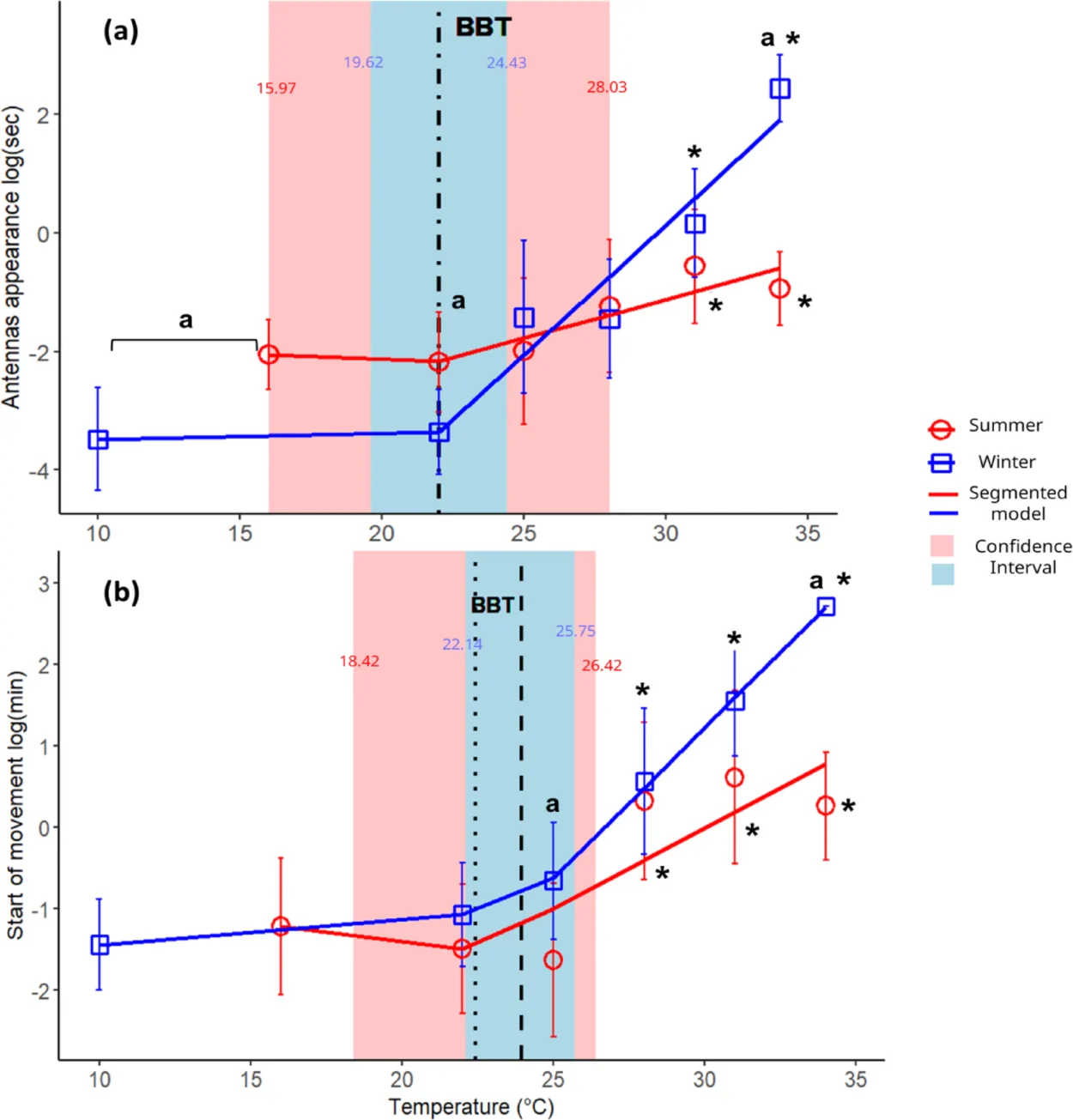
Behavioural response of the periwinkle *Littorina littorea* to acute warming (1.5 °C/h) for winter and summer groups starting at their specific control temperature. **a** Time for snails to extend their antennae. **b** Time for snails to initiate movement. Data are mean ± standard deviation (N = 10). Asterisks indicate significant differences from the respective control temperature within a group, while ‘a’ indicates significant differences between seasonal groups at the same temperature. Behavioural breakpoint temperatures (BBTs) are indicated by a dashed line for the winter group and a dotted line for the summer group. Coloured areas represent the confidence interval 95% (C.I.) of the BBT, with numbers indicating the temperature limits of the C.I

Snails’ behavioral response was affected by the increase in temperature, with the reaction time for extending their antennae and beginning to move increasing with warming in both winter and summer snails. The winter snails reacted stronger to the temperature increase as indicated by a steeper increase in the response time (Fig. 2).

Up to 22 °C, both winter and summer snails showed a consistent time to extend their antennae with further warming, response times progressively increased (Fig. 2a). The behavioral breakpoint temperature (BBT), was 22.02 °C (95% CI 19.62–24.43; p value = 4.4e−9; R2 = 0.79) and 22.00 °C (95% CI 15.97–28.03; p value = 0.042; R2 = 0.26) for the winter and summer snails, respectively. Summer snails showed higher individual variability in this response as shown by the wider confidence interval (by 7 °C) in summer compared to winter group. At 31 °C, both winter and summer snail reacted significantly slower than at their respective control temperatures (Z = − 4.09, *p* = 5.69 × 10^−4^; summer: Z = − 3.71, *p* = 0.003). Comparison between the two seasons revealed significant differences, winter snails responded faster than summer snails at temperatures ≤ 22 °C (16 °C: W = 22.5, *p* = 0.002; 22 °C: W = 16, *p* = 0.02) but slower at 34 °C (W = 100, *p* = 9.8e-4).

For the start of the movement (Fig. 2b), we found different BBTs between the groups, with two degrees higher BBT in the winter group (winter BBT: 23.94 °C, 95% CI 22.14–25.75, p value = 2.8e-12, R2 = 0.85; summer BBT: 22.42 °C, 95% CI 18.42–26.42, p value = 0.0226, R2 = 0.38). The confidence interval for the BBT of the summer group was wider (by 4 °C) than in the winter group. Beyond the BBT, the time required for snails to initiate movement increased and at 28 °C, both groups reacted significantly slower than at their respective control temperatures (winter: Z = − 3.15, *p* = 0.001; summer: Z = − 3.28, *p* = 0.01). Between groups significant differences were found at 25 °C (W = 86.5, *p* = 0.02) and 34 °C (W = 100, *p* = 3.8e−4), with winter snails initiating movement later than summer snails at both temperatures.

### Metabolites

**Fig. 3:**
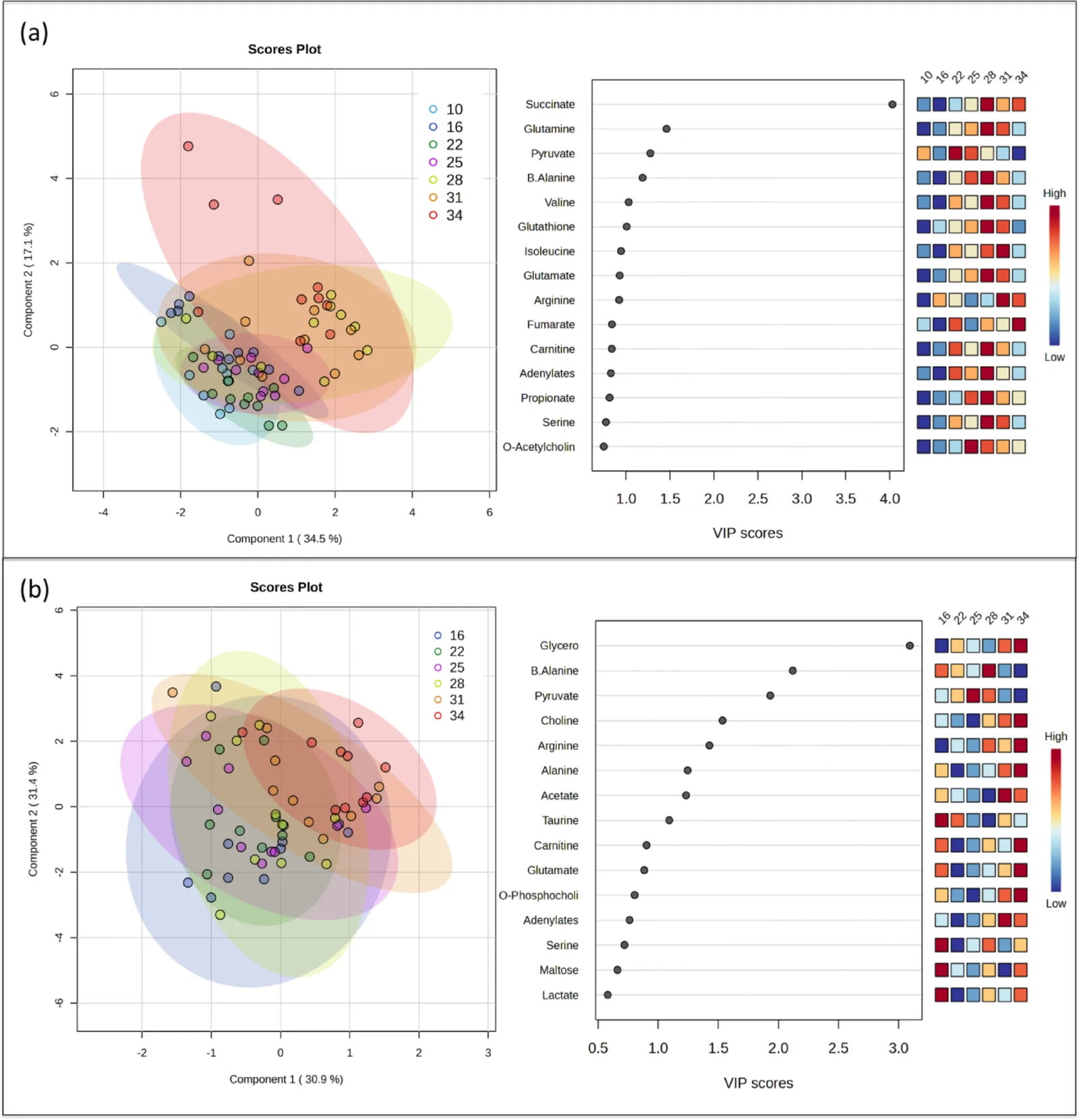
Effect of temperature on metabolite profiles measured in the total soft body tissue of *Littorina littorea* exposed to acute warming (1.5 °C/h) in winter (**a**) and summer (**b**) groups, starting at their specific control temperature. Score plots (left) and variable importance in projection (VIP, right) scores of the partial least-square discriminant analysis (PLS-DA). Coloured circles in the score plots represent individual samples at respective temperatures. The VIP score shows the weighted sum of squares of the PLS loadings. The colour-coded boxes next to the VIP scores reflect the concentrations of the respective metabolite per temperature (*N* = 10 per temperature)

We identified 34 metabolites in whole soft body tissue of *L. littorea*, including amino acids and derivatives (alanine, valine, isoleucine, leucine, glutamate, glutamine, arginine, methionine, serine and β-alanine), osmolytes (betaine, taurine, homarine and trimethylamine N-oxide), energy related compounds (acetate, ADP, ATP, AMP, carnitine, lactate, pyruvate, succinate, fumarate, malate, and propionate), signaling/neurotransmission (sn-glycero-3-phosphocholine, choline, O-phosphocholine, O-acetycholine, and 4-aminobutyrate), antioxidants (glutathione and hypotaurine) and carbohydrates (glucose and maltose). Figure 3 shows the metabolite profiles of the winter and summer snails sampled at different temperatures in the plane of two first PLS-DA components. In the summer group, there was no clear separation of samples according to temperature, whereas in the winter group, metabolite profiles of the snails were clearly separated between low (10–16 °C) and high (31–34 °C) temperatures. Variable Importance in Projection (VIP) scores identified the metabolites contributing most to group separation, those with VIP scores > 1 were considered the most influential in discriminating between temperatures.

**Fig. 4:**
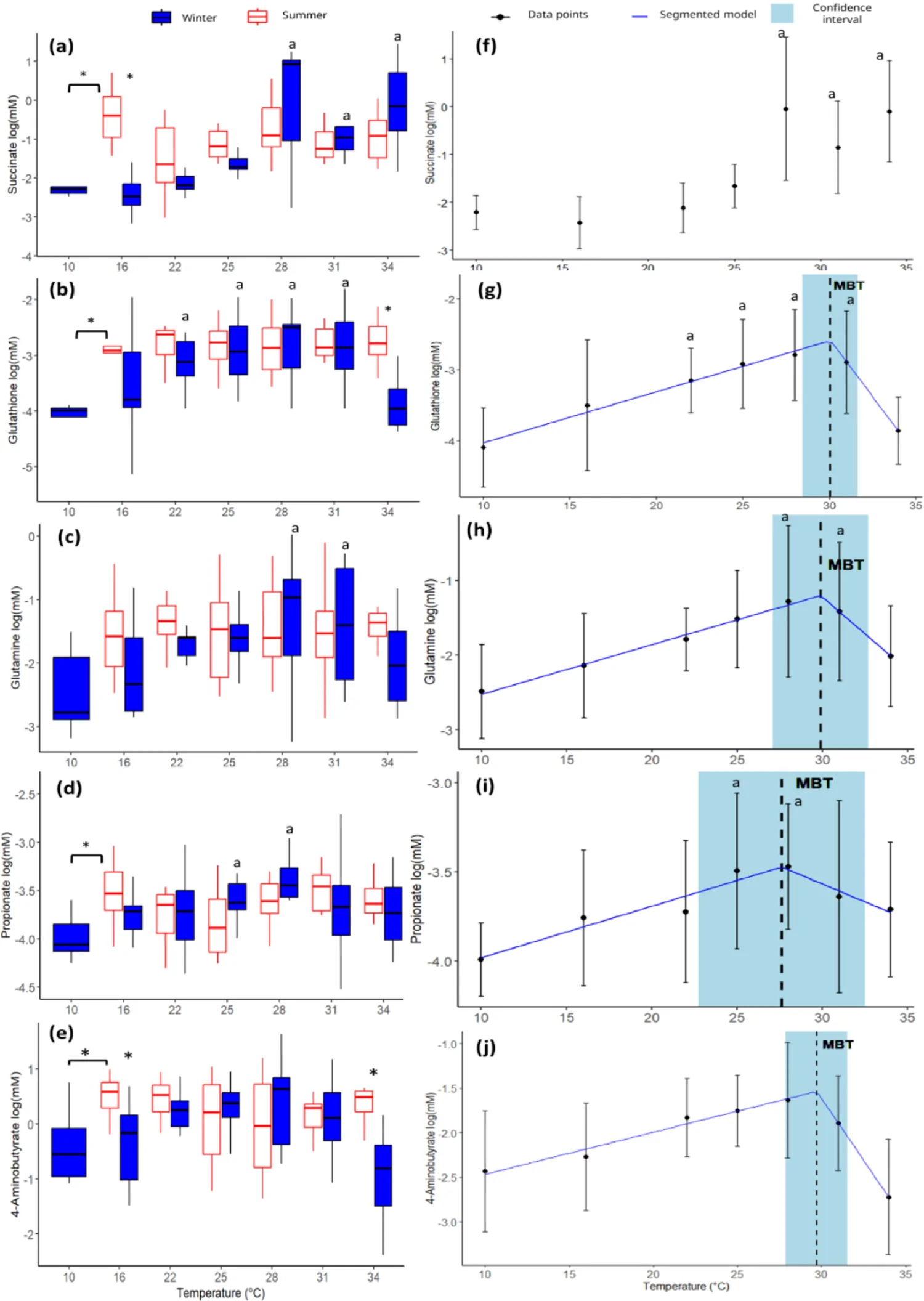
Concentrations of selected metabolites measured in total soft body tissue of *Littorina littorea* in winter and summer groups exposed to acute warming (1.5 °C/h) starting at their specific control temperature. **a** Succinate. **b** Glutathione. **c** Glutamine. **d** Propionate. **e** 4-Aminobutyrate. **f**–**j** Representation of the segmented models for these metabolites and the corresponding metabolite breakpoint temperatures (MBT) determined in the winter group only. Data are **a**–**e** box plots with median (bold line) and **f**–**j** mean ± standard deviation (N = 10). Asterisks indicate significant differences from the respective control temperature within a group, while ‘a’ indicates significant differences between seasonal groups at the same temperature

In winter snails, progressive warming led to a linear increase in the concentrations of various metabolites including succinate, glutathione, glutamine, propionate, and 4-aminobutyrate. The metabolites changed significantly at specific temperatures (Kruskal–Wallis; *p* < 0.05, except 4-aminobutyrate) and were also identified as variables of importance in our SAM (FDR < 0.05) and PLS-DA (VIP > 1) analyses (see Fig. 4 and Supplementary Table 1). Pyruvate and taurine were also identified as important variables in the winter group, but no significant differences between temperatures were detected (see Supplementary Fig. 8). In contrast, none of the 34 metabolites identified in the summer group showed significant temperature-dependent changes in concentration. PLS-DA indicated low predictive ability (Q² ≤ 0.11; R² = 0.60; *p* = 0.02), suggesting potential overfitting. Consistently, SAM analysis (FDR-adjusted p value > 0.05) did not identify any discriminating variables. Figure 4 shows the warming-induced changes in concentration of those metabolites that followed a linear increase in concentration with temperature with their corresponding metabolic break point temperature (MBT). Snails of the winter group showed a significant increase in succinate concentration (Fig. 4a) at temperatures ≥ 28 °C (28 °C: Z = − 3.59, *p* = 0.003; 31 °C: Z = − 2.99, *p* = 0.02; 34 °C: Z = − 3.93, *p* = 0.0008). The observed temperature-dependent changes in succinate concentrations with consistently elevated levels above 28 °C made it difficult to calculate the MBT (Fig. 4f). Although succinate concentration in the summer group was not affected by acute warming, summer snails had significantly higher concentrations than winter snails at their respective control temperatures (W = 10, *p* = 0.01).

Glutathione concentrations in the winter group increased linearly with warming, with significantly elevated levels at 22 °C (Z = − 2.84, *p* = 0.03), 25 °C (Z = − 3.56, *p* = 0.004), 28 °C (Z = − 3.87, *p* = 0.001), and 31 °C (Z = − 3.51, *p* = 0.004; Fig. 4b). The MBT for glutathione was 30.05 °C (95% CI 28.45–31.64 °C; R² = 0.34; *p* = 8.4e−6; Fig. 4g). Significant seasonal differences in the glutathione content were found at control temperatures (W = 1, *p* = 0.002) and 34 °C (W = 3, *p* = 0.002), with higher concentrations observed in summer snails. Glutamine concentrations in the snails from the winter group increased with warming, reaching significantly elevated concentrations at 28 °C (Z = − 3.32, *p* = 0.009) and 31 °C (Z = − 2.92, *p* = 0.03), before returning to baseline levels at 34 °C (Fig. 4c). The MBT for glutamine was 29.89 °C (95% CI 27.08–32.71 °C; R² = 0.21; *p* = 0.007; Fig. 4h). Glutamine concentrations appeared lower in the winter group at control temperatures, 22 °C and 34 °C; these differences were not statistically significant. Similarly, propionate concentrations in the winter group increased linearly with warming, with significant elevations detected at 25 °C (Z = − 2.92, *p* = 0.04) and 28 °C (Z = − 3.29, *p* = 0.01; Fig. 4d). The MBT was 27.62 °C (95% CI 22.73–32.51 °C; R² = 0.11; Fig. 4i) but there was no significant change in the slope (David Test, *p* = 0.06). A significant difference between seasons was observed at control temperatures, with higher concentrations in the summer group (W = 12, *p* = 0.03). 4-Aminobutyrate concentrations in winter snails showed an increasing trend with warming at 28 °C and a decrease at 34 °C, although these changes were not statistically significant (Fig. 4e). The MBT was 29.71 °C (95% CI 27.86–31.57 °C; R² = 0.28; *p* < 0.001; Fig. 4j). Significant differences between seasons were observed at the control temperature (W = 17, *p* = 0.04) and 34 °C (W = 7, *p* = 0.009), with lower concentrations found in winter snails.

### Pathway analysis

Pathway analysis in the winter group revealed that at temperatures above 22 °C a series of metabolic pathways significantly changed compared to 10 °C. At 25 °C, the antioxidant pathway (via glutathione) and at 28 °C, a broader set of pathways, including the TCA cycle and additional amino acid metabolisms (alanine, aspartate, and glutamate), were altered. At 31–34 °C, these trends persisted without the emergence of additional affected pathways. For specific metabolites affected in each pathway, together with impact values, see the supplementary materials (Supplementary Table 2). There were no significant changes in any pathway in the summer snails.

### Heat shock proteins and enzyme activity

**Fig. 5:**
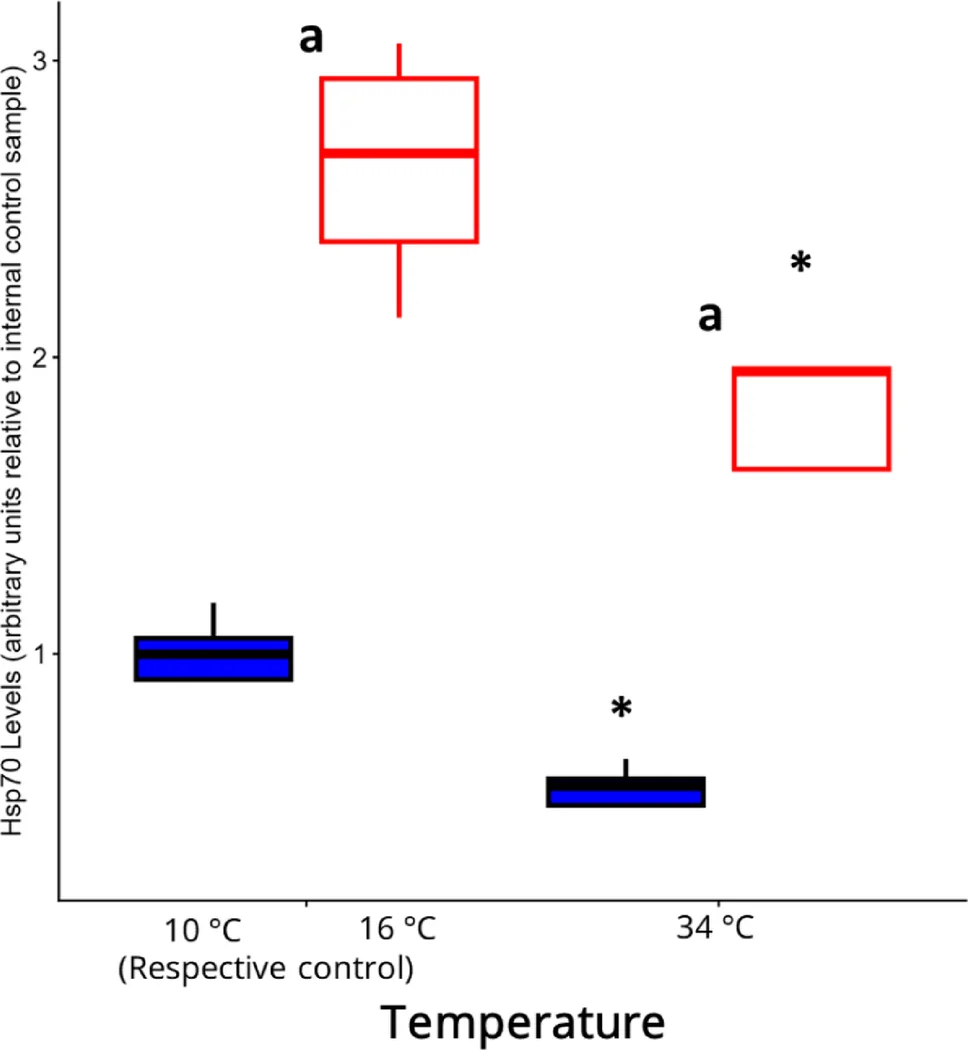
Hsp70 levels in total soft body tissue of the winter and summer group of *L. littorea* measured at the respective control temperature and 34 °C after acute warming of 1.5 °C/h. Data are mean ± standard deviation (N = 4). Asterisks indicate significant differences from the respective control temperature within a group, while ‘a’ indicates significant differences between seasonal groups at the same temperature

Hsp70 levels measured at the respective control temperatures (10 °C and 16 °C) and the final common temperature (34 °C) varied significantly within and between seasonal groups (Fig. 5). After acute warming to 34 °C, Hsp70 levels decreased significantly in both groups compared to their respective controls, with a decrease of 47% in the winter (t=-3.8, df = 5.5, p value = 0.012) and 38% in the summer group (t = − 2.6, df = 5.1, p value = 0.047). At both temperatures measured, the comparison between groups revealed nearly 3-times higher Hsp70 levels in the summer than the winter snails (t = 5.0, df = 9.1, p value = 7.5e−4).

**Fig. 6:**
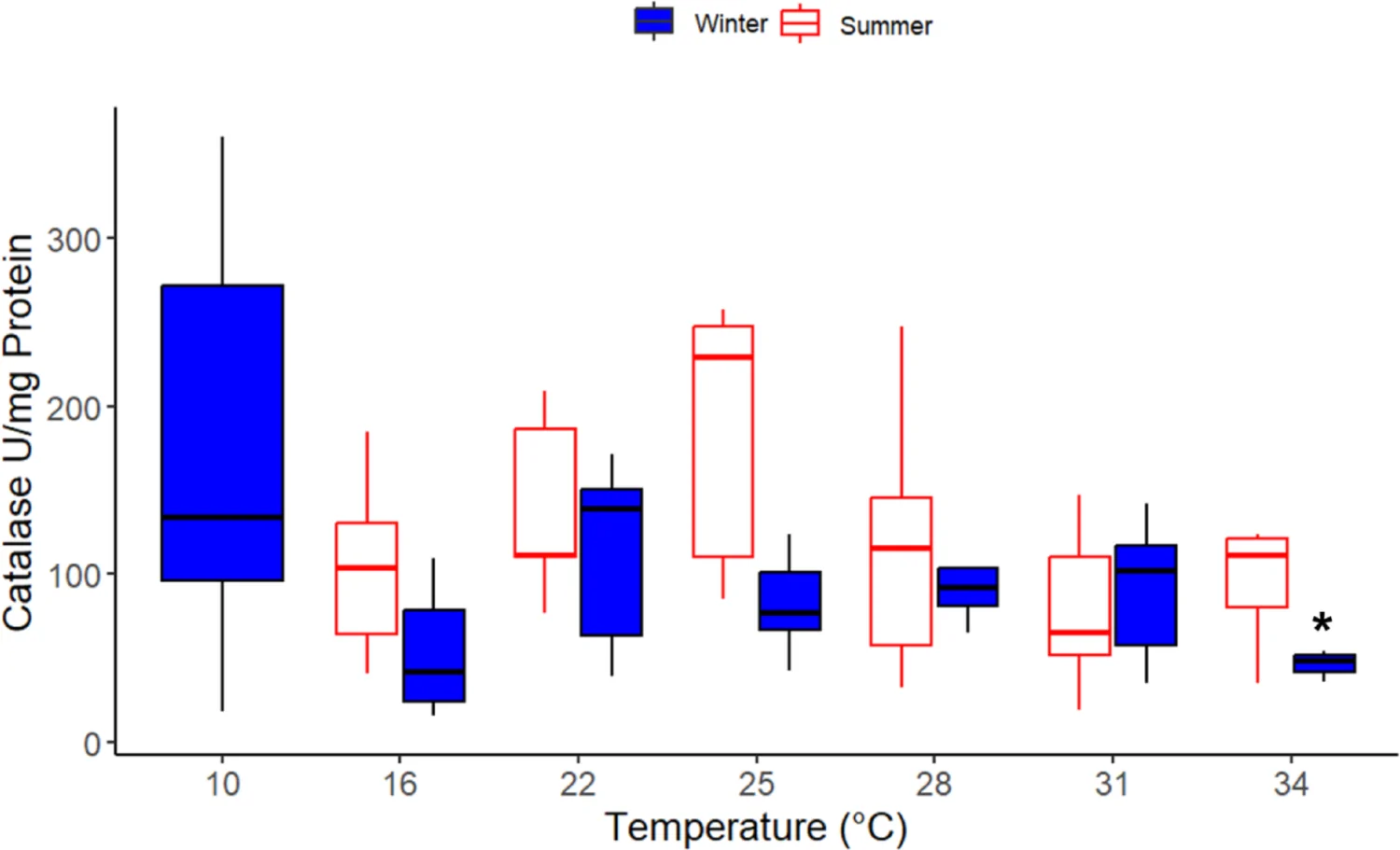
Catalase activity measured at a common temperature (25 °C) in total soft body tissue of *Littorina littorea* in winter and summer groups exposed to acute warming (1.5 °C/h) starting at their respective control temperatures. Data are mean ± standard deviation (*N* = 5–7). Significant differences from the respective control temperature within the winter group are indicated with an asterisk

The summer group exhibited higher CAT activity than the winter group at all temperatures tested but these differences were not significant (Fig. 6). Within the summer group, CAT activity tends to increase with exposure temperature, peaking at 25 °C, followed by a decline at higher temperatures. In contrast, CAT activity in the winter group decreased during acute warming but then remained relatively stable across different warmer temperatures. CAT activity in winter snails sampled at control temperature (10 °C) showed high variability and was significantly higher than in the snails sampled at 34 °C (Z = 2.94, *p* = 0.03).

## Discussion

### Pejus and critical temperatures

In the present study, the season-dependent thermal sensitivity of *L. littorea* was determined based on the upper sublethal thermal thresholds, pejus (Tp) and critical (Tc) temperatures. Studies have used different approaches to determine such thermal thresholds using proxies at the organismal, cellular and molecular levels (Sartoris et al. 2003; Klok et al. 2004; Lannig et al. 2004; Nilsson et al. 2009; Windisch et al. 2014; Giomi et al. 2019; Eymann et al. 2020; Götze et al. 2020; Pörtner et al. 2017; Frederich and Lancaster 2024). The behavioural responses of ectotherms to temperature were shown to be a reliable whole-organism proxy to determine thermal thresholds and can be used to analyze thermal performance curves (TPCs) as locomotor activity typically increases with warming up to an optimum, after which it decreases as thermal stress increases (Monaco et al. 2017); i.e. .the swimming activity was used in Tp determination of Atlantic cod, *Gadus morhua* (Schakmann et al. 2023). The behavioral response of *L. littorea* remained stable up to a certain temperature (BBT), after which it gradually slowed down with increasing temperature. Based on the temperature-dependent time-to-antenna extension and time-to-movement start, the Tp was similar for the winter and summer groups, ranging from 22 to 24 °C. The warming-induced reduction of movement of *L. littorea* is consistent with patterns observed in other marine species. For example, Peck et al. (2004) exposed individuals of the clam *Laternula elliptica* and the limpet *Nacella concinna* to an acute temperature ramp (0.1 °C/h) with a maximum temperature of 5 °C after acclimation at 0 °C. For both species, the authors found a significant decrease in the proportion of individuals able to burrow and right themselves with increasing temperature. Schram et al. (2014) assessed righting response in Antarctic *N. concinna* and *Margarella antarctica* exposed for six weeks to different combinations of temperature and pH, and found that exposure to high temperatures (3.5 °C) and low pH (7.8) inhibited the righting response. Some studies have linked behavioural changes to physiological adaptations at the organismal level. For example, Jost et al. (2012) determined the upper Tp and Tc evaluating the righting response, heart rate, lactate concentration and the activity of AMP-activated protein kinase (AMPK) in three decapod species *Cancer irroratus*, *Carcinus maenas* and *Homarus americanus* exposed to acute warming (6 °C/h). Thermal tolerance in these species correlated with their effective thermal habitat niche, and the upper temperatures inhibiting the righting response was 30 °C, 31 °C, and 36 °C for *C. irroratus*, *C. maenas* and *H. americanus*, respectively. At these temperatures, a decrease in heart rate and increased lactate levels were observed indicating the onset of anaerobic metabolism at Tc. Anestis et al. (2007) studied the behavioral (valve closing behavior) and physiological (pyruvate kinase activity) responses of the mussel *Mytilus galloprovincialis* to acute warming (18–31 °C at 0.1 °C/min) and found a direct relationship between temperature increase and valve closure time where the behavioral change was associated with a reduction in pyruvate kinase activity and a decrease in overall energy turnover, reinforcing the idea that behavioural responses to thermal stress are closely linked to metabolic processes.

We observed more pronounced changes in *L. littorea* behavior and metabolism in winter compared to summer snails. While this association does not imply a cause-and-effect relationship, it suggests that seasonal acclimatization influences the sensitivity of various behavioral, physiological, and metabolic processes to warming. Although the BBT for behavior was similar in both summer and winter groups (22 °C), the decline in behavioral response to stimuli was much more pronounced in winter snails above this threshold. In addition to an increase in behavioral response time at temperatures above 25 °C, alterations in the metabolism of glutathione, an important antioxidant, indicates the onset of oxidative stress and elevated ROS levels in winter snails (Giannetto et al. 2017). At 28 °C, the alterations of the TCA cycle, and several amino acid pathways indicate a broader response involving both metabolic substrate usage and cellular defense mechanisms. At 31 °C and 34 °C, continued changes in the mentioned metabolic pathways, along with disruption of the TCA cycle, indicate that increasing thermal stress causes systemic metabolic disruption involving antioxidant defense, energy production, and protein/amino acid turnover. It is important to note that the aforementioned metabolic changes, along with the other molecular endpoints, provide a snapshot of the organism’s state at a given temperature and time, and do not reflect kinetics.

Activation of molecular chaperones, such as Hsp70, and the antioxidant system are a most significant part of general cellular stress response, and play a key role in survival of intertidal species in a challenging habitat (Kultz 2005; Somero 2002, 2020; Dong et al. 2008). Hsp can bind to and stabilize unfolded or aggregated proteins (Mayer and Bukau 2005), and the antioxidant enzyme catalase (CAT) catalyzes the decomposition of hydrogen peroxide to water and oxygen (Chainy et al. 2016). We found seasonal variation in the activity of CAT and the levels of Hsp 70 (see “Seasonal Differences” section below), but only a modest impact of acute warming on these parameters. In the present study, CAT activity measured in the whole soft tissue of *L. littorina* remained largely unchanged under acute warming across both seasonal groups. As CAT is a part of a complex, multi-component antioxidant system (AOS), the absence of response to warming can be associated with a possible trade-off of different components of AOS, like other AOS enzymes and antioxidant compounds. Indeed, change in the CAT activity in different organisms under the different warming scenario appears to be largely diverse. For example, CAT activity increased in whole soft tissue of terrestrial snails *Xeropicta derbentina* (25/38/40 °C vs. 43/45 °C) by Troschinski et al. (2014) and in gill tissue of the mussel *Mytilus galloprovincialis* (15 °C vs. 30/35 °C) by Wang et al. (2018). In fish, muscle CAT activity either increased, decreased or remained unaffected after acute warming dependent on investigated species (Madeira et al. 2012). Similarly, more prolonged warming (up to 30 days) revealed diverse responses in invertebrates with increased CAT activity in mussel whole soft tissue at 25 °C compared to 10/15 °C (Khessiba et al. 2005); decreased or unchanged activity in mussel gill and digestive gland at 24 °C compared to 20 °C (Vieira et al. 2021) or in clam whole soft tissue at 22 °C compared to 17.5 °C (Crespo et al. 2021). Khan et al. (2021) reported an increase in SOD but not CAT activity in gill tissue of the mussel *Mytilus coruscus* during warming (20 vs. 30 °C). These findings highlight the species- and tissue-specific responses of antioxidant systems including catalase, which plays a key role in the integrated cellular defense against warming-induced oxidative stress.

Compared to controls, acute warming resulted in significantly lowered Hsp70 levels at 34 °C, in both summer and winter groups of *L. littorina*. Although we lack measurements at intermediate temperatures, reduced Hsp70 levels at 34 °C indicate that exposure to extreme temperatures can overwhelm this protective mechanism, particularly as 34 °C approaches the reported heat coma threshold for *L. littorea* at 35 °C (Clarke et al. 2000), a point at which physiological functions are compromised. A similar suppression of Hsp70 levels at extreme temperatures was observed in *X. derbentina*, where Hsp70 levels increased up to their maximum expression at 40 °C followed by a decrease at higher temperatures (Troschinski et al. 2014).

An increase in the levels of anaerobic end products such as succinate, acetate, or propionate indicates the onset of anaerobiosis and, in the context of warming, transition to the critical temperature range (De Zwaan and Wusman 1976; Grieshaber et al. 1994; Müller et al. 2012; Götze et al. 2020, 2025; Georgoulis et al. 2022). We observed significant succinate accumulation in winter snails at temperatures of 28 °C and above, whereas no similar temperature-dependent accumulation was observed in summer snails. Warming-induced succinate accumulation has been reported in *L. littorea*, *L. saxatilis*, and *Echinolittorina malaccana* (Melatunan et al. 2011; Sokolova and Pörtner 2003; Chen et al. 2021). Interestingly, in the eastern oyster *Crassostrea virginica*, the critical temperature threshold (Tc), indicated by succinate accumulation, shifted to higher levels in spring (28 °C) compared to winter (24 °C; (Bagwe et al. 2015). A similar seasonal shift in Tc may explain the absence of succinate accumulation in the summer-collected *L. littorea* at the tested elevated temperatures.

We also observed a gradual accumulation of propionate during warming, but only in winter snails, reaching up to approximately 28 °C. Propionate is a final byproduct of anaerobic metabolism in mollusks, including mussels, where it is formed through the reduction of succinate, typically under prolonged anaerobic conditions (Müller et al. 2012). The accumulation of propionate supports the hypothesis that, during acute warming, *L. littorea* may transition to partially anaerobic ATP production, a response similar to that observed during long-term heat stress in *M. galloprovincialis* (Feidantsis et al. 2020). In *M. galloprovincialis*, succinate accumulates during the early phase of exposure, followed by a decline and a subsequent increase in propionate during prolonged heat stress and anoxia (Isani et al. 1995; Feidantsis et al. 2020). Similarly, in a long-term anaerobiosis study, *Halicryptus spinulosus*, *Astarte borealis*, and *Arctica islandica* showed progressive accumulation of both succinate and propionate, with a species-specific delay in propionate increase (Oeschger 1990). Given the acute temperature ramp used in our study, the succinate-to-propionate conversion pathway may not fully explain the observed propionate accumulation and further research is required to determine the cause of propionate accumulation. Nevertheless, given its role in energy metabolism, the observed temperature-dependent increase in propionate likely reflects altered energy production and may result from anaerobiosis induced by thermal stress.

A decline in glutamine concentrations above 28 °C observed in winter snails in our present study may be linked to increased energy demand and the onset of anaerobic metabolism in response to heat stress. Glutamine can be converted to α-ketoglutarate, which supports both the electron transport chain for aerobic energy production and anaerobic metabolism, including the conversion of aspartate to oxaloacetate and eventually succinate, thereby aiding ATP production through multiple pathways during heat stress (Müller et al. 2012; Ericson et al. 2022). Furthermore, in addition to its metabolic role, glutamine is a key precursor of glutamate, an excitatory neurotransmitter. Glutamate can then be decarboxylated to 4-aminobutyrate (GABA; (Albrecht et al. 2010; Walls et al. 2015), a major inhibitory neurotransmitter in mammals that regulates neuronal excitability through specific receptors and transporters (Shelp et al. 2017). In gastropods, GABA has also been implicated in the regulation of feeding behaviour (Miller 2019). The decline in glutamine was accompanied by a decrease in GABA concentrations at 34 °C in the winter group, on one hand it could reflect reduced feeding activity at high temperatures or, more generally, a sign of overall metabolic dysregulation at extreme thermal stress.

Glutathione is an intracellular antioxidant that scavenges reactive oxygen species (ROS) and reactive nitrogen species (RNS) and provides reducing equivalents to antioxidant enzymes such as glutathione peroxidase, which detoxifies peroxides. This activity preserves the integrity of lipids, proteins, and nucleic acids under both normal and stress conditions (Abele and Puntarulo 2004; Marí et al. 2009; Farina and Aschner 2019). The function of glutathione is well conserved from an evolutionary perspective (Hewitt and Degnan 2023), and its accumulation at high temperatures is a common response in both ectotherms (Abele and Puntarulo 2004; Leggatt et al. 2007; Leeuwis and Gamperl 2022; Papadopoulos et al. 2025) and endotherms (Mitchell et al. 1983; Russo et al. 1984; Edwards et al. 2001; Surai et al. 2016; Sejian et al. 2018) as a compensatory response to ROS accumulation (Marí et al. 2009; Aquilano et al. 2014).

Taken together, our data on behavioral and biochemical changes suggest that the upper critical temperature (Tc) for winter snails is approximately 28 °C, while the pejus temperature (Tp) is around 22 °C. In the summer group, Tp was approximately 22 °C, albeit with a broad confidence interval, whereas no Tc could be determined based on the accumulation of anaerobic end products suggesting a shift in Tc to higher values beyond the tested temperature range. The thermal limits determined for *L. littorea* in our study suggest that the Helgoland population falls within its thermal tolerance under historical climate conditions. Winter and summer sea surface temperatures (SST) are generally below the Tp and Tc thresholds, with averages of 5.2 °C (maxima 10.2–14.2 °C; 1982–2023) and 16 °C (maxima 16.4–21.3; 1982–2023), respectively (OISST V2.1). However, as an intertidal species, *L. littorea* often experiences significant daily temperature fluctuations, including extreme conditions on rocky surfaces or in rock pools (Morris and Taylor 1983). For example, a heatwave occurred in the North Sea in 2018 during which sea surface temperatures reached 21 °C, approaching the Tp identified here (Kaiser et al. 2023). Moreover, it has been reported that temperatures over 30 °C can occur in isolated rock pools during low tides in Helgoland (Gimenez, personal communication, June 4, 2024). Despite these harsh conditions, behavioral adaptations such as foot retraction, aggregation, and refuge selection allow *L. littorea* to reduce internal temperatures by several degrees (Ng et al. 2017). For example, Miller and Denny (2011) found that foot retraction and shell reorientation can reduce body temperature by 2–4 °C in littorinid snails (*Littorina keenae*, *L. scutulata*, *L. plena*, *L. sitkana*, and *Echinolittorina natalensis*), and Hayford et al. (2021) demonstrated that the intertidal snail *Nucella ostrina* exhibit behavioral thermoregulation through selective use of microhabitats. Such phenomena allow mobile invertebrates to remain below physiological thresholds even when external temperatures are extreme and warrants greater consideration in climate change research. In light of increasing heatwave frequency, duration and intensity and the general trend of global warming (IPCC 2023) the proposed thresholds might have ecological implications dependent on the adaptive capacity of the species.

### Seasonal differences

The seasonal comparison revealed greater sensitivity to acute warming in winter than summer snails, as reflected in their stronger behavioral response and the onset of anaerobiosis indicating an upper Tc of 28 °C. Shifts in upper Tc depending on seasonal acclimatization or laboratory-based acclimation are well-documented e.g., Sommer et al. (1997) and Wittmann et al. (2008) and have also been observed in *Littorina* species. For instance, *L. saxatilis* from the North Sea exhibited a lower upper Tc of 15 °C in cold-acclimated snails (4 °C) compared to 28 °C in those acclimated to warmer temperatures (13 °C; (Sokolova and Pörtner 2003). Similarly, in the freshwater mussel *Dreissena polymorpha*, Tp (determined by AMPK activation) increased with rising environmental temperatures across seasons, from 16 °C (winter, 8 °C acclimated) to 24 °C (spring, 16 °C acclimated) and 32 °C (summer, 24 °C acclimated; (Jost et al. 2015).

In our present study, the baseline Hsp70 levels were higher in summer than winter snails and remained elevated during acute exposure to 34 °C. Similarly, CAT activity basal levels tended to be higher in the summer compared to the winter group. Increased Hsp levels and CAT activity may contribute to the higher thermal tolerance observed in the summer group. Seasonal variation in the levels of antioxidants and molecular chaperones has also been documented in other invertebrates. For example, Kong et al. (2008) found that CAT activity in the gills of the mud crab *Scylla serrata* was higher in summer than winter animals. Protein expression of CAT, SOD and Hsp70 in gills of the American oyster *Crassostrea virginica* was upregulated with increasing ambient temperatures (Rahman and Rahman 2021). Similarly, seasonal monitoring of Hsp70 levels in the land snail *Xeropicta derbentina* (Dieterich et al. 2013) or in the gills of the intertidal mussel *Mytilus trossulus* (Hofmann and Somero 1995) revealed highest expression during summer. In contrast, Delorme et al. (2020) reported that, while acclimation to 24 °C increased Hsp70 mRNA expression in the sea urchin *Evechinus chloroticus* compared to an acclimation at 18 °C, this molecular response did not result in a higher upper thermal limit. Additionally, the authors did not find seasonal differences in thermal tolerance. Overall, these findings suggest that elevated Hsp70 levels do not universally result in enhanced thermal tolerance at the whole-organism level and point to possible species-specific or mechanistic differences.

In addition to metabolic changes, differences in thermal tolerance may also be influenced by the reproductive cycle of the species. Although capable of reproducing throughout the year, *L. littorea* exhibits seasonal changes, such as shedding of the male penis during summer in association with testis regression (Barroso et al. 2007). Moreover, Williams (1970) observed that lipid and carbohydrate reserves are accumulated in summer (feeding/growth phase) and utilised in winter, when gonad maturation and peak reproductive activity occur. These life-history characteristics suggest that winter individuals may prioritise energy investment in reproduction over thermal resilience, which could contribute to their lower thermal tolerance compared to summer animals.

In summary, *Littorina littorea* exhibited seasonally distinct behavioural and metabolic, and cellular responses to acute warming. Winter snails showed a progressive metabolic imbalance with succinate accumulation, amino acid remodeling, and activation of antioxidant defenses, consistent with a shift towards anaerobiosis, while summer snails exhibited attenuated responses, likely reflecting higher baseline protection through elevated Hsp70 and catalase activity. From these responses we identified sublethal thresholds within the OCLTT framework: Tp of ~ 22 °C for both, winter and summer snails associated with declining performance and metabolic adjustments, and Tc of 28 °C (winter snails only), marked by a transition to anaerobic metabolism. Given that the present Tp estimates were derived exclusively from behavioral responses with broad confidence intervals (18–28 °C for winter snails and 16–28 °C for summer snails), additional whole-animal physiological metrics, such as heart rate and oxygen consumption, would likely provide more sensitive indicators of thermal stress. Nevertheless, present findings highlight the importance of understanding the seasonal variability of Tp and Tc in ectotherms, as this is crucial for more accurate predictions and modeling of ecosystem resilience in the context of climate change. With the increasing frequency of heatwaves across all seasons—including warmer winters and potential winter heatwaves—this knowledge is vital for assessing future ecological impacts.

## Supplementary Information

Below is the link to the electronic supplementary material.

Supplementary Material 1

## Data Availability

The datasets generated and analysed during the current study are available in PANGEA (after publication)
