# Supplementary material for: Seasonal impact on the thermal tolerance window of a keystone coastal grazer, the common periwinkle Littorina littorea
Source: J Comp Physiol B. 2026 May 29;196(4):409–27. doi: 10.1007/s00360-026-01670-3 (PMC13506667; doi:10.1007/s00360-026-01670-3)
Supplement: Supplementary file 1 [file 360_2026_1670_MOESM1_ESM.docx]

Seasonal impact on the thermal tolerance window of a common coastal inhabitant, the periwinkle *Littorina littorea*

Journal: Journal of Comparative Physiology B Biochemical, Systems, and Environmental Physiology

Authors:

Tomás A. García (0009-0008-5533-5912)^1*^, Daria Bedulina (0000-0001-7650-0974)^1^, Inna Sokolova (0000-0002-2068-4302)^2,3^, Gisela Lannig (0000-0002-9210-256X)^1^

Affiliation:

^1^Department of Integrative Ecophysiology, Alfred-Wegner-Institute Helmholtz Centre for Polar and Marine Research, Bremerhaven, Germany

^2^Department of Marine Biology, Institute for Biological Sciences, University of Rostock, Rostock, Germany

^3^Department of Maritime Systems, Interdisciplinary Faculty, University of Rostock, Rostock, Germany

Corresponding author:

*tomas.garcia@awi.de

# Supplementary Material

**Fig. 7** Representative Western blot images of Hsp70 protein expression in total soft body tissue of *Littorina littorea* exposed to control temperatures and to 34 °C following acute warming (1.5 °C h⁻¹), shown for (A) the winter and (B) the summer group. On the left side of each image is the molecular weight marker, with a red rectangle enclosing the samples and the 70 kDa band of the marker. Numbers represent individual samples of the same treatment. Winter: control T = 10°C (1-4) vs. 34°C (5-8). Summer: control T = 16°C (9-12) vs. 34°C (13-16). I.C. indicates internal control. The image was obtained with the LAS-1000 imaging system (Fujifilm, Japan).

**Table 1** Important features identified by SAM for the temperature dependent changes in metabolite profiles determined in total soft body tissue of *Littorina littorea* of the winter group. SAM test statistics representing differences between temperatures (d.value), variability of metabolite concentration (stdev), uncorrected probability (rawp) and FDR-corrected p-value (q.value), is shown.

| Compounds | d.value | stdev | rawp | q.value |
| --- | --- | --- | --- | --- |
| Succinate | 13.64 | 0.26 | 0 | 0 |
| Glutathione | 6.32 | 0.23 | 0 | 0 |
| Pyruvate | 5.81 | 0.49 | 0.0003 | 0.001 |
| 4-Aminobutyrate | 4.92 | 0.22 | 0.001 | 0.003 |
| Taurine | 3.68 | 0.30 | 0.01 | 0.01 |
| Glutamine | 3.44 | 0.29 | 0.01 | 0.01 |

**Fig. 8** Concentrations of pyruvate and taurine measured in total soft body tissues of *Littorina littorea* in winter and summer groups exposed to acute warming (1.5 °C/h) starting at their specific control temperature. Data are presented as box plots with median (bold line) and mean ± standard deviation with N=10 in mM (a-b) and after log10 transformation and pareto scaling (c-d). Significant differences from the respective control temperature within the winter group are indicated by 'a'.

**Table 2** Major metabolic pathways identified by pathway analysis contributing to temperature dependent changes in metabolite profiles determined in total soft body tissue of *Littorina littorea* of the winter group exposed to acute warming (1.5 °C/h). "Hits" refer to the number of metabolites in our dataset that match the compounds within a given pathway. –Log₁₀(p) represents the negative logarithm of the p-value; only pathways with values below 2.5 were included. The p-value for pairwise comparisons between the control temperature (10 °C) and the respective elevated temperature is shown for affected metabolite. "Impact" quantifies the relative importance of a metabolic pathway, based on both enrichment and the topological position of the metabolites identified in that pathway. High pathway impact score indicates that the perturbations found in that pathway involve metabolites that are structurally or functionally central to the pathway's biological network, whereas low impact scores suggest that although metabolites may be statistically significant, they occupy peripheral positions in the pathway network. Therefore, pathways with impact scores below 0.1 were excluded to focus on functionally relevant metabolic alterations.

| Temperature | Pathway | Total compounds | Hits | -Log10(p) | Affected metabolites | p.value | Impact |
| --- | --- | --- | --- | --- | --- | --- | --- |
| 25 | Glutathione metabolism | 28 | 2 | 2.9 | Glutathione | <0.001 | 0.28 |
|  |  |  |  |  | L-Glutamate |  |  |
| 28 | Glutathione metabolism | 28 | 2 | 2.8 | Glutathione | <0.001 | 0.28 |
|  |  |  |  |  | L-Glutamate | <0.1 |  |
|  | Citrate Cycle (TCA cycle) | 20 | 3 | 2.7 | Pyruvate |  | 0.11 |
|  |  |  |  |  | Fumarate |  |  |
|  |  |  |  |  | Succinate | <0.001 |  |
|  | Alanine, aspartate and glutamate metabolism | 27 | 7 | 2.5 | L-Alanine |  | 0.47 |
|  |  |  |  |  | Pyruvate |  |  |
|  |  |  |  |  | Fumarate |  |  |
|  |  |  |  |  | L-Glutamate | <0.1 |  |
|  |  |  |  |  | 4-Aminobutyrate | <0.05 |  |
|  |  |  |  |  | Glutamine | <0.05 |  |
|  |  |  |  |  | Succinate | <0.001 |  |
| 31 | Glutathione metabolism | 28 | 2 | 2.8 | Glutathione | <0.001 | 0.28 |
|  |  |  |  |  | L-Glutamate | <0.1 |  |
| 34 | Citrate Cycle (TCA cycle) | 20 | 3 | 2.7 | Pyruvate |  | 0.11 |
|  |  |  |  |  | Fumarate | <0.1 |  |
|  |  |  |  |  | Succinate | <0.001 |  |
|  | Alanine, aspartate and glutamate metabolism | 27 | 7 | 2.5 | L-Alanine | <0.1 | 0.47 |
|  |  |  |  |  | Pyruvate |  |  |
|  |  |  |  |  | Fumarate | <0.1 |  |
|  |  |  |  |  | L-Glutamate |  |  |
|  |  |  |  |  | 4-Aminobutyrate |  |  |
|  |  |  |  |  | Glutamine |  |  |
|  |  |  |  |  | Succinate | <0.001 |  |
